# Development of a recombinant replication-deficient rabies virus-based bivalent-vaccine against MERS-CoV and rabies virus and its humoral immunogenicity in mice

**DOI:** 10.1371/journal.pone.0223684

**Published:** 2019-10-07

**Authors:** Hirofumi Kato, Mutsuyo Takayama-Ito, Itoe Iizuka-Shiota, Shuetsu Fukushi, Guillermo Posadas-Herrera, Madoka Horiya, Masaaki Satoh, Tomoki Yoshikawa, Souichi Yamada, Shizuko Harada, Hikaru Fujii, Miho Shibamura, Takuya Inagaki, Kinjiro Morimoto, Masayuki Saijo, Chang-Kweng Lim

**Affiliations:** 1 Department of Virology I, National Institute of Infectious Diseases, Shinjuku-ku, Tokyo, Japan; 2 Department of Life Science and Medical Bioscience, Waseda University, Shinjuku-ku, Tokyo, Japan; 3 Department of Pharmacy, Yasuda Women’s University, Hiroshima, Hiroshima, Japan; Instituto Butantan, BRAZIL

## Abstract

Middle East respiratory syndrome-coronavirus (MERS-CoV) is an emerging virus that causes severe disease with fatal outcomes; however, there are currently no approved vaccines or specific treatments against MERS-CoV. Here, we developed a novel bivalent vaccine against MERS-CoV and rabies virus (RV) using the replication-incompetent P-gene-deficient RV (RVΔP), which has been previously established as a promising and safe viral vector. MERS-CoV spike glycoprotein comprises S1 and S2 subunits, with the S1 subunit being a primary target of neutralizing antibodies. Recombinant RVΔP, which expresses S1 fused with transmembrane and cytoplasmic domains together with 14 amino acids from the ectodomains of the RV-glycoprotein (RV-G), was developed using a reverse genetics method and named RVΔP-MERS/S1. Following generation of RVΔP-MERS/S1 and RVΔP, our analysis revealed that they shared similar growth properties, with the expression of S1 in RVΔP-MERS/S1-infected cells confirmed by immunofluorescence and western blot, and the immunogenicity and pathogenicity evaluated using mouse infection experiments. We observed no rabies-associated signs or symptoms in mice inoculated with RVΔP-MERS/S1. Moreover, virus-specific neutralizing antibodies against both MERS-CoV and RV were induced in mice inoculated intraperitoneally with RVΔP-MERS/S1. These findings indicate that RVΔP-MERS/S1 is a promising and safe bivalent-vaccine candidate against both MERS-CoV and RV.

## Introduction

Middle East respiratory syndrome (MERS) is a highly lethal respiratory disease caused by a single-stranded, positive-sense RNA betacoronavirus, the MERS-coronavirus (MERS-CoV) [1,2]. The severity of MERS ranges from asymptomatic or mild disease to acute respiratory distress syndrome leading to death. Clinical features include fever, cough, shortness of breath, and multi-organ failure resulting in death, especially in individuals with underlying comorbidities, such as diabetes and renal failure [1]. Since MERS-CoV was first isolated from a patient with fatal respiratory disease in the Kingdom of Saudi Arabia in 2012 [3], the World Health Organization (WHO) has been notified of >2,300 laboratory confirmed cases of MERS-CoV infection and >800 deaths as of April 2019 [4]. Although MERS occurs in the Middle East, including the Kingdom of Saudi Arabia and the United Arab Emirates, patients with MERS have also been reported from MERS non-endemic regions, such as Europe, the United States, and Asia, as imported cases from the Middle East. Additionally, a large outbreak in South Korea suggested that MERS remains a serious threat to global public health [5].

Vaccination is expected to be an efficacious strategy in preventing individuals and animals from suffering MERS-CoV infections. To date, various kinds of candidate vaccines for MERS have been developed, including live attenuated, subunit, DNA, prime-boost, and recombinant vector vaccines [6,7]; however, no approved vaccine or specific treatment for MERS is currently available. MERS-CoV spike glycoprotein comprises S1 and S2 subunit regions, with the S1 subunit of MERS-CoV responsible for its binding to host cells expressing the viral receptor dipeptidyl peptidase 4 through the receptor-binding domain (RBD) [8–10]. During vaccine development, previous studies showed that the S1 protein could serve as a dominant target for virus-specific neutralizing antibodies (VNAs) [11–13]. In fact, S1 proteins have been used as the antigen in several MERS-CoV vaccine preparations. For example, full-length S protein or truncated S1-subunit glycoprotein has been incorporated into several vectored vaccines against MERS-CoV, subsequently eliciting VNAs following inoculation of these candidates into animals [14,15].

Rabies is a viral disease caused by rabies virus (RV), which is a negative-sense, single-stranded RNA virus of the *Rhabdoviridae* family with a simple genome organization encoding five structural proteins [16]. Rabies in humans is almost always fatal upon the appearance of clinical symptoms; however, rabies is a vaccine-preventable disease, with rabies-inactivated vaccines providing close to 100% protection by pre- or post-exposure prophylaxis and having saved millions of lives since the development of the first rabies vaccination for humans in 1885 [17]. An estimated 55,000 people still die of rabies annually, with cases reported from >150 countries and territories among various animals (mainly dogs) and humans; therefore, the WHO has set a goal to eliminate human deaths due to rabies by 2030 [18]. Inactivated rabies vaccines are currently available worldwide; however, they are not an ideal strategy because they require frequent administrations (4–6 doses). On this point, attenuated live vaccines represent a promising and attractive alternative, because they can elicit both humoral and cellular immunity [19], suggesting that frequent vaccinations are not needed.

The RV genome encodes five structural proteins: nucleoprotein (N), phosphoprotein (P), matrix protein (M), glycoprotein (G), and large protein (L) [16]. P-gene-deficient rabies virus (RVΔP) is replication-incompetent, as the RV-P protein is a multi-functional protein that serves to stabilize the RV-L protein, a major component of the viral RNA polymerase [20–22]. Similarly, M-gene-deficient RV (RVΔM) is propagation impaired, because the RV-M protein participates in the budding process [23]. RVΔP and RVΔM remain capable of expressing viral proteins, assembling, and propagating only in specially constructed cells expressing the RV-P and RV-M proteins, respectively; however, infectious progeny of RVΔP and RVΔM are not produced in other cells. Therefore, these viruses are considered candidates for developing safe and effective attenuated RV vaccines for humans and animals [24–26].

RV represents an attractive expression vector with potential utility as a viral vaccine vector [27–29] based on several advantages for vaccine development [30,31]: 1) RV possesses a relatively simple, modular genome organization, rendering its genetic modification easier relative to other complex genomes of DNA and plus-stranded RNA viruses [32,33]; 2) RV exhibits a cytoplasmic replication cycle, suggesting that the virus would not affect the genome of host cells [27,34]; 3) the full-genome RV vector provides sufficient capacity to insert large foreign genes that can be stably expressed and preserved [28]; 4) RV is non-cytopathic in infected cells, thereby allowing sustained production of an inserted gene over extended periods [34–36]; 5) RV can induce a protective immune response in a variety of mammalian species [37]; and 6) pre-existing vector immunity to RV does not prevent the induction of antibodies against a foreign antigen, suggesting that an RV-based on vaccination strategy might be effective in previously RV-vaccinated humans, and that boosting with various RV-vectored vaccines might be successful [38].

Based on these advantages, several bivalent vaccines against other targeted diseases using replication-incompetent RV harboring foreign genes inserted by reverse genetic techniques have been developed. In this study, we developed a bivalent-vaccine candidate against MERS-CoV and RV using RVΔP as a vector and evaluated the induction of a humoral immune response to MERS-CoV and RV, as well as its safety profile.

## Materials and methods

### Cells

Neuro-2a (N2A) cells were obtained from the JCRB cell bank (IFO50081), originating from the American Type Culture Collection (ATCC; #CCL-131; Manassas, VA, USA). Vero cells and 293T cells were purchased from the ATCC (#CCL-81 and #CRL-3216, respectively). N2A cells and 293T cells were grown in Dulbecco’s modified Eagle medium (DMEM; Sigma-Aldrich, St. Louis, MO, USA) supplemented with 10% heat-inactivated fetal bovine serum (FBS; Biowest, Nuaillé, France) and antibiotics (DMEM-10FBS; 100 U/mL penicillin and 100 μg/mL streptomycin; Thermo Fisher Scientific, Waltham, MA, USA). Vero cells were grown in DMEM supplemented with 5% heat-inactivated FBS and antibiotics (DMEM-5FBS). BHK-21 cell lines expressing recombinant RV-P protein (BHK-P) were grown in DMEM-10FBS and 200 μg/mL Zeocin (Thermo Fisher Scientific) [26].

### Generation of RVΔP expressing the MERS-CoV S1 spike glycoprotein

**Fig 1:**
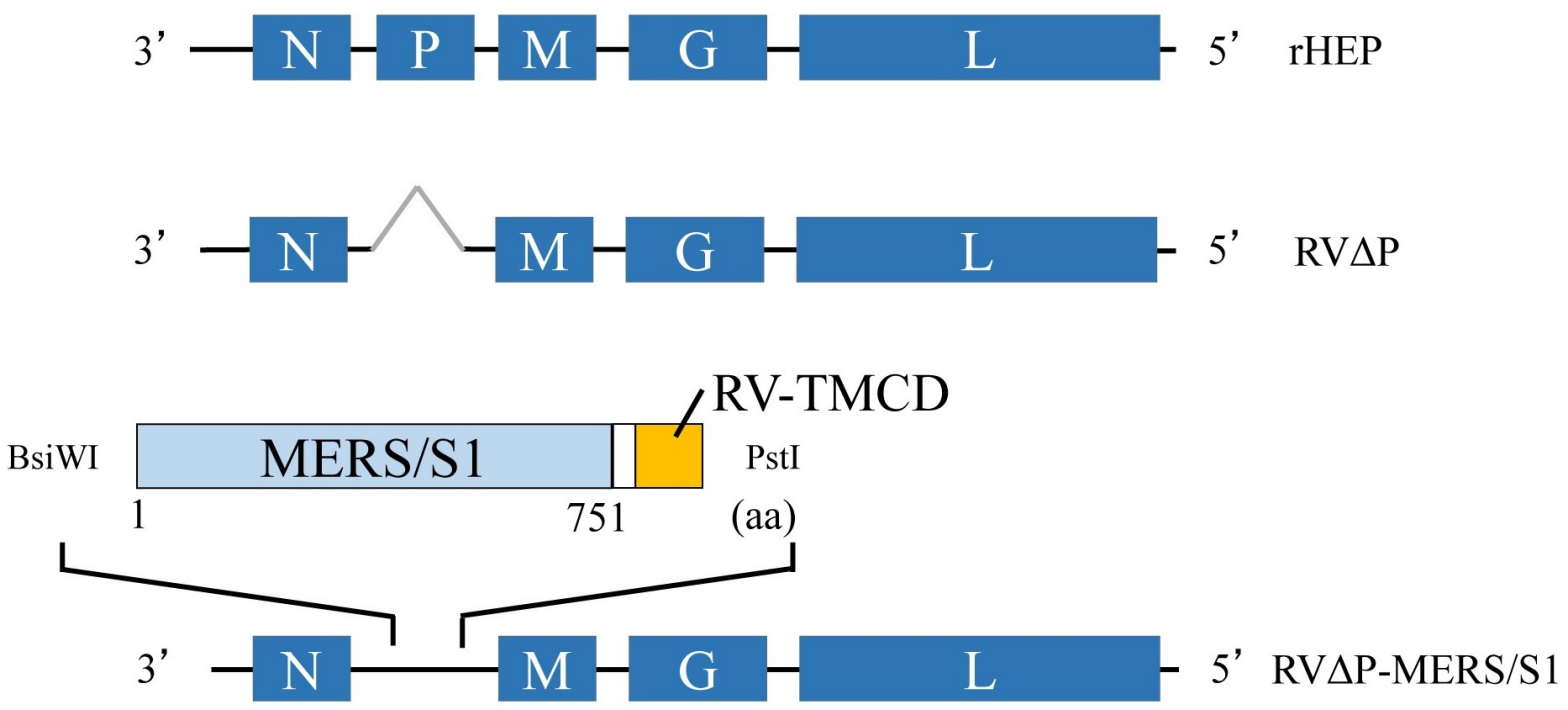
Schematic illustration of recombinant RV genome constructs used in this study. Recombinant HEP-Flury (rHEP) has a complete genome of RV HEP-Flury strain (upper). RVΔP lacks the RV-P gene (middle). RVΔP-MERS/S1 harbors the MERS-CoV S1 gene fused with the C-terminal region of RV G protein (amino acids 446 to 524), which includes transmembrane, cytoplasmic domain, and stem domains of RV-G gene between RV-N and RV-M genes of the genome (lower).

MERS-CoV spike glycoprotein comprises S1 and S2 subunit regions, with the S1 subunit a primary target of VNAs. The S protein was cleaved into the S1 and S2 subunits by various host proteases [2]. To express the S1 protein efficiently on the membrane of the virion, RV-G transmembrane and cytoplasmic domains were fused with the S1 protein. cDNA encoding the RV-G transmembrane and cytoplasmic domains together with 14 amino acids of the ectodomain of RV-G (RV/TMCD) was amplified with primers harboring *Bsi*WI and *Pst*I restriction enzyme sites at the 5′ ends, followed by cloning of the pGEM-T Easy vector (Promega, Madison, WI, USA). The RV/TMCD fragment was digested and inserted into the *Bsi*WI and *Pst*I site of plasmid p3.1-defP [26], resulting in p5.1-defP-RV/TMCD. The cDNA region corresponding to amino acids 1 through 751 of the codon-optimized S glycoprotein (Sino Biological, Beijing, China) of MERS-CoV EMC/2012 (accession number: AFS88936.1) was amplified and inserted into plasmid p5.1-defP-RV/TMCD using the In-Fusion HD cloning kit (Takara Bio, Shiga, Japan) to produce p5.1-defP-MERS/S1-RV/TMCD (Fig 1).

### Viruses

HEP-Flury is the parental strain of RVΔP (GenBank: AB085828.1) and has been applied for human use in an inactivated RV due to its status among the most attenuated RV strains. HEP-Flury causes no symptoms in adult mice but kills suckling mice when inoculated intracerebrally [39]. A recombinant HEP virus (rHEP) was rescued from full-length cDNA of the HEP-Flury strain and propagated in BHK cells [40]. RVΔP was rescued from full-length cDNA of the RV genome lacking the RV-P gene and four helper plasmids (pH-N, pH-P, pH-G, and pH-L) by a reverse genetics method and propagated in BHK-P cells, as described previously [26]. Additionally, RVΔP-MERS/S1 was rescued from 293T cells using the reverse genetics method from p5.1-defP-MERS/S1-RV/TMCD and the four helper plasmids, followed by propagation in BHK-P cells.

For the animal experiments, RVΔP and RVΔP-MERS/S1 were purified and concentrated. Briefly, BHK-P cells were infected with RVΔP-MERS/S1 or RVΔP at a multiplicity of infection (MOI) of between 0.05/cell and 0.5/cell and maintained at 33 °C in hyper flasks (Corning, Corning, NY, USA) filled with DMEM supplemented with 2% FBS and antibiotics (DMEM-2FBS). The supernatant was collected on days 7, 14, and 21, and the culture medium was replaced on days 7 and 14. The supernatant was filtered using 0.45-μm polyethersulfone membrane filters (Thermo Fisher Scientific) to remove cell debris, and the released viruses were concentrated by precipitation with 7% polyethylene glycol (PEG) 6000 (WAKO, Osaka, Japan). Virions were purified by ultracentrifugation of the precipitate for 90 min at 83,000 *g* with 60% and 20% sucrose (WAKO) and treated with Amicon Ultra-15 (Merck Corporation, Darmstadt, Germany). Purified virus stocks were stored at −80 °C until use.

### Immunofluorescence assay

BHK-P cells were seeded onto culture plates, and on the following day infected with RVΔP or RVΔP-MERS/S1 at 37 °C for 1 h. After replacing the inoculum with fresh DMEM-2FBS, cells were cultured at 33 °C for 48 h. Cells were subsequently fixed with Mildform10N (WAKO) for 20 min and washed twice with phosphate-buffered saline (PBS). Cells were permeabilized in 0.5% Triton X-100 for 20 min at room temperature and washed with PBS three times. To detect MERS-CoV S1 expression, cells blocked in PBS containing 2% FBS for 1 h were then stained with mouse monoclonal antibody (mAb) against MERS-CoV S1 [45E11; kindly provided by Dr. Kazuo Ohnishi from the National Institute of Infectious Diseases (NIID)] [41] at 37 °C for 1 h. Cells were washed with PBS then reacted with the secondary antibody Dylight 549-conjugated polyclonal anti-mouse immunoglobulin (Ig)G (H+L) (Vector Laboratories, Burlingame, CA, USA). To detect RV-N expression, cells were stained with fluorescein isothiocyanate (FITC)-labeled anti-RV mAb (Fujirebio, Tokyo, Japan) at 37 °C for 1 h. Fluorescent images were observed using a confocal laser-scanning microscope (FluoView FV3000; Olympus, Tokyo, Japan).

### Virus titration

Titers of RVΔP and newly generated RVΔP-MERS/S1 were determined by focus assay in BHK-P cells, as described previously [29]. Briefly, BHK-P cells prepared in 96-well plates the previous day were inoculated with each virus solution diluted 10-fold serially and incubated at 33 °C for 3 days. Thereafter, the fixed cells with 80% acetone at room temperature for 20 min were stained with FITC-labeled anti-RV mAb. The foci were counted under a fluorescence microscope.

### Western blot

Cells infected with either of RVΔP-MERS/S1 or RVΔP were incubated at 33°C for 48 h, followed by washing with PBS twice, lysis, and centrifugation at 12,000 *g* at 4°C for 10 min. Sample supernatants were mixed with an equal volume of sample buffer containing 2-mercaptoethanol and incubated at 98°C for 2 min. Samples were separated using precast 10% gels for sodium dodecyl sulfate-polyacrylamide gel electrophoresis (SDS-PAGE; ATTO, Tokyo, Japan) and transferred onto polyvinylidene difluoride membranes, which were placed in an iBind western system (Thermo Fisher Scientific) according to manufacturer instructions. The primary antibody used to detect RV-G was anti-RV-G mAb15-13 [42] kindly provided by Dr. Nobuyuki Minamoto (Gifu University, Gifu, Japan). The primary antibody used to detect the MERS-CoV spike protein was a polyclonal antibody against MERS-CoV spike protein S1 (Sino Biological). Additionally, we used a polyclonal antibody against tubulin (Medical & Biological Laboratories, Nagoya, Japan) to detect tubulin as the internal control. Secondary antibodies for the detection of RV-G and the MERS-CoV spike protein were horseradish peroxidase-conjugated anti-mouse and anti-rabbit IgG (H+L) (Thermo Fisher Scientific), respectively. After incubation with the secondary antibodies for 2.5 h, membranes were washed with deionized water, stained with SuperSignal West Femto (Thermo Fisher Scientific), and visualized using a LAS-3000 (Fujifilm, Tokyo, Japan). The MERS-CoV spike S1 protein (Sino Biological) was used as a positive control.

### Safety profile test

One-day-old ICR suckling mice were purchased from Japan SLC (Shizuoka, Japan) and allowed to acclimate for 3 days. Eight suckling mice were placed in a cage with their untreated mother. Cages were randomly divided into three groups, and suckling mice were injected with RVΔP-MERS/S1, RVΔP, or rHEP [each group included two cages (*n* = 16 mice]. All mice were inoculated intracerebrally with 20 μL of each virus solution containing 10^7^ focus-forming units (FFU)/mL. The suckling mice were observed for clinical signs for 3 weeks.

### Immunization test

Three-week-old female BALB/c mice were purchased from Japan SLC and allowed to acclimate for 1 week. Mice were randomly divided into five experimental groups, as shown in Table 1. All mice were inoculated intraperitoneally with 100 μL of each virus solution containing 10^7^ FFU/mL or PBS, with the day on which mice were inoculated with viruses defined as day 0. Blood samples were collected by cardiac puncture under terminal anesthesia with isoflurane on day 14 or 28, and all mice were euthanized after blood collection. Experiments were performed in duplicate.

**Table 1:** Experimental mouse groups in the immunization test.

Group	Viruses	No. of inoculation	No. of mice
1	RVΔP-MERS/S1	1	5
2	RVΔP-MERS/S1	2	6
3	RVΔP	1	5
4	RVΔP	2	5
5	PBS	2	5

### MERS-CoV-neutralizing test

Sera were separated from whole-blood samples by centrifugation and stored at −20°C until required. Before testing, sera were heated at 56°C for 30 min to inactivate complement factors. A neutralization test on live MERS-CoV was performed, as described previously [43,44]. Briefly, serially diluted serum samples were mixed with virus solution containing 50 plaque-forming units of MERS-CoV (EMC isolate). After 1 h, Vero cells seeded on 96-well culture plates were inoculated with each of the serum-virus mixtures [43]. At 5- or 7-days post-infection, the cells were fixed with 10% formalin and stained with crystal violet. Cytopathic effects (CPEs) on Vero cells were observed, and the neutralization titer was determined as the highest dilution that showed at least 50% CPE inhibition.

### RV-neutralizing test

Titers of VNAs against RV were determined using a modified rapid fluorescent focus-forming inhibition test, as described previously [45,46]. Briefly, the heat-inactivated sera described in the previous subsection were serially diluted 2-fold, and 50 μL sera was added to each well of the 96-well cell culture plates. These sera were mixed with an equal volume of HEP-Flury virus solution containing a 50% focus-forming dose. After incubation at 37 °C for 1 h, suspended N2A cells were added to the wells (2.0 ×10^5^ cells/mL; 50 μL/well), and cells were incubated at 37 °C for 48 h and then fixed with 80% acetone for 20 min at room temperature. Cells were then stained with the FITC-labeled anti-RV antibody at 37°C for 1 h. The VNA titer was defined as serum dilution at 50% fluorescent focus reduction in the infected cultures. The 50% neutralization dose was calculated according to the Spearman–Kärber method, and values were normalized to international units (IUs) using WHO anti-RV Ig.

### Statistical analysis

The Mann–Whitney *U* test and log-rank test were used to compare viral growth and compare characteristics between fatal and nonfatal mice in the Kaplan–Meier curves, respectively. The Steel–Dwass nonparametric test was used for multiple comparisons of VNA titers. All p-values were two-sided, and a p < 0.05 was considered significant. All data were analyzed using STATA for Windows (v.13.1; StataCorp LP, College Station, TX, USA) or JMP software (v.11.0; SAS Institute, Cary, NC, USA).

### Animal ethics statement

All animal studies were performed in strict accordance with recommendations described in the Guidelines for Proper Conduct of Animal Experiments of the Science Council of Japan and strict compliance with animal husbandry and welfare regulations. All animal experiments were reviewed and approved by the Institutional Animal Care and Use Committee of the NIID (approval Nos. 117042 and 117043). All animals infected with RV were handled in biosafety level 2 animal facilities in accordance with NIID guidelines. Mice were inoculated with virus under proper anesthesia.

### Humane endpoints

In this study, we used humane endpoints as early indicators of animal pain or distress that could be used to avoid or limit suffering by taking actions such as humane euthanasia. During the observation period, we monitored neurological symptoms daily and set up the humane endpoint when mice were considered to have reached a moribund stage [i.e., observation of rabies-associated clinical signs after infection (e.g., paralysis or seizure)]. Moribund mice were euthanized with isoflurane immediately after they reached endpoint criteria. All research staff were specially trained in animal care and treatment under the standard operation procedures of our laboratory.

## Results

### Construction of RVΔP-MERS/S1 and expression of viral proteins

**Fig 2:**
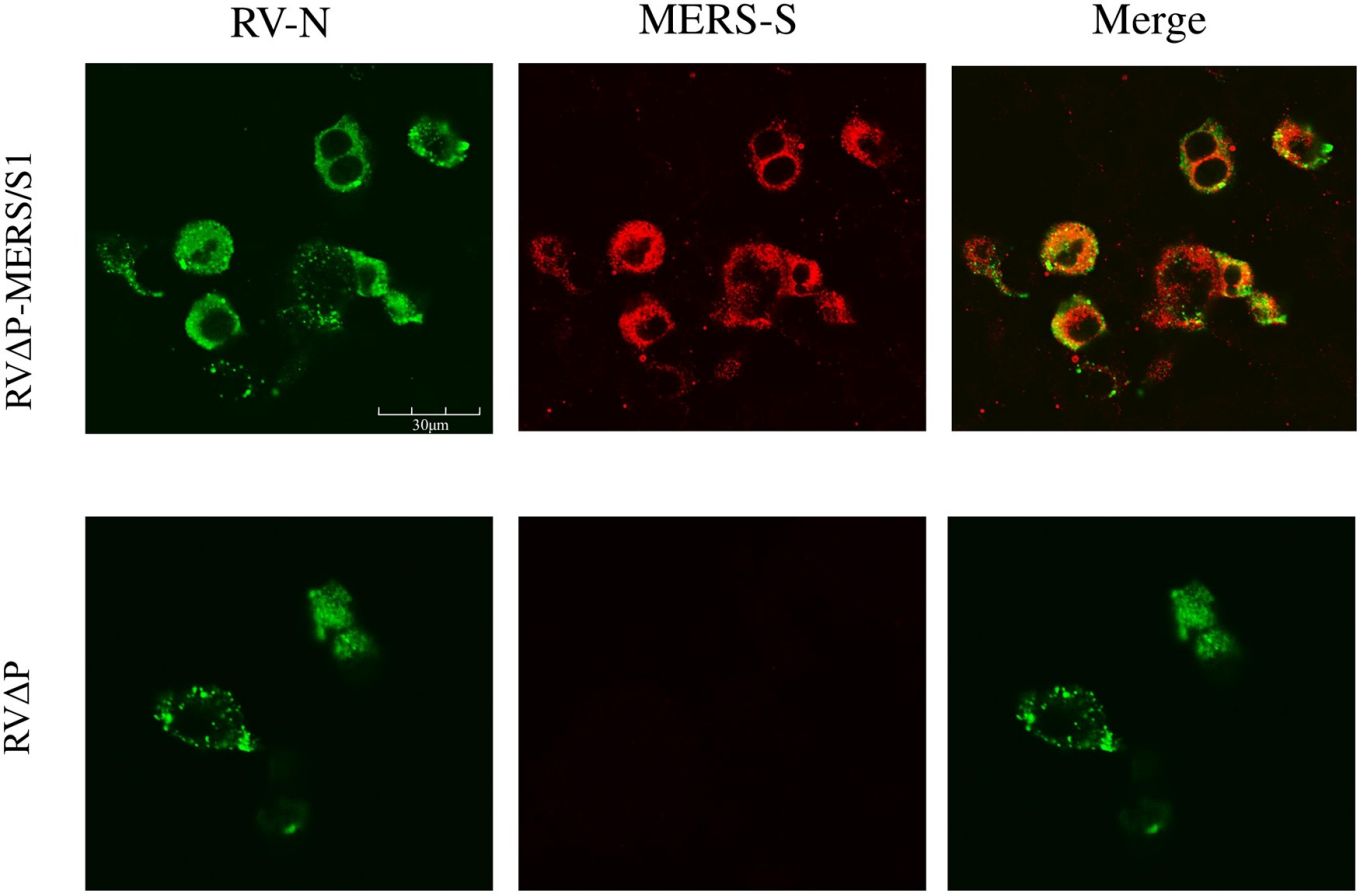
Immunofluorescence staining of RV-N and MERS-CoV S1 protein expression. BHK-P cells were inoculated with either RVΔP-MERS/S1 or RVΔP at an MOI of 0.1/cell and incubated at 33 °C for 48 h. Cells were stained with the monoclonal antibody against RV-N (green) or the monoclonal antibody MERS-CoV S1 protein (red), respectively.

**Fig 3:**
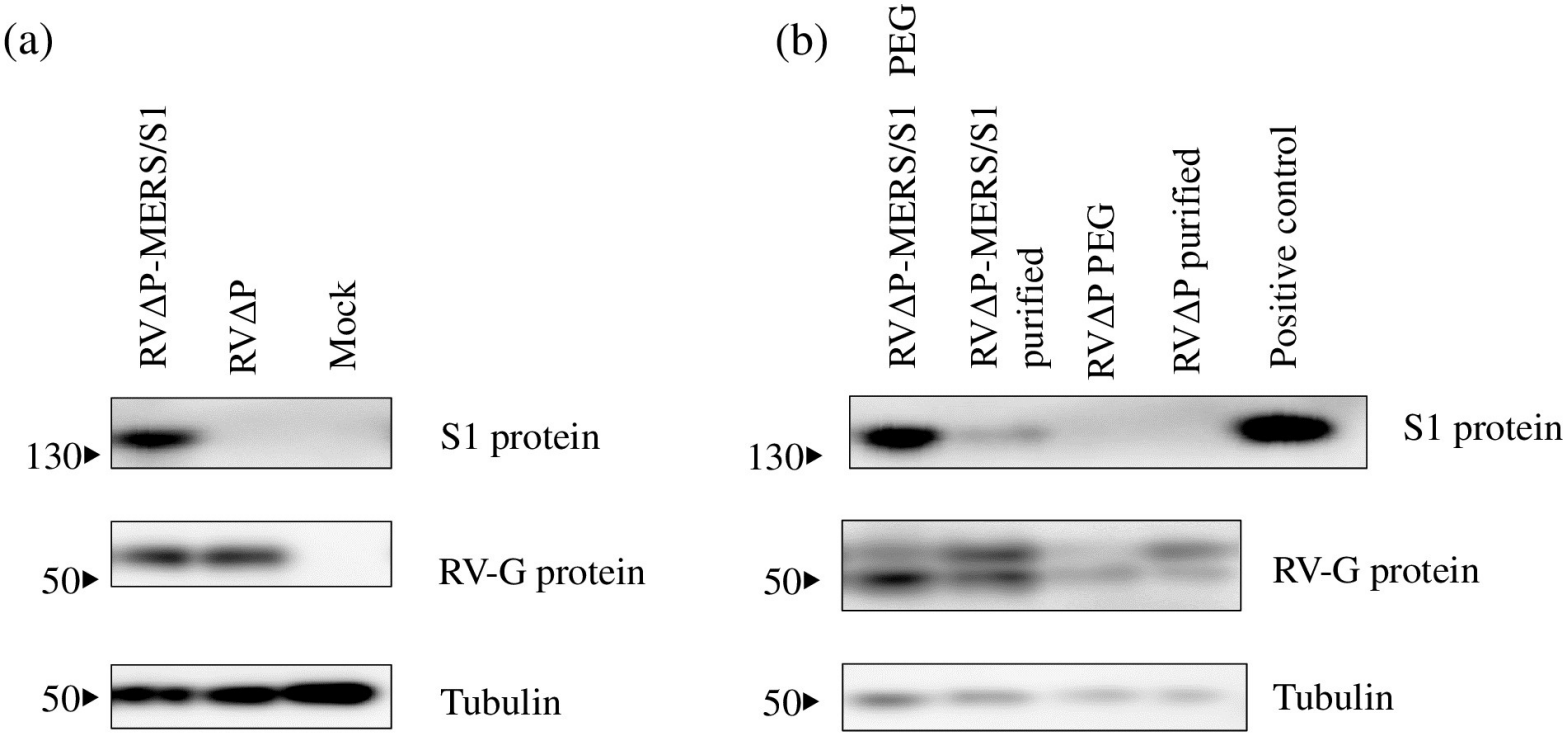
Western blotting analysis of RV-G and MERS-S1 protein expression. BHK-P cells were inoculated with RVΔP-MERS/S1 or RVΔP and incubated at 33 °C for 48 h. RV-G protein and MERS-S1 protein expression were confirmed with western blotting using monoclonal antibody against RV-G and polyclonal antibody against MERS-CoV S1 protein, respectively, a) in cell lysate preparations and b) in polyethylene glycol (PEG)-precipitated or sucrose-purified viruses. Recombinant MERS-CoV S1 protein were used as a positive control.

Recombinant viruses (RVΔP-MERS/S1 and RVΔP) were generated from plasmid p5.1-defP-MERS/S1-RV/TMCD using a reverse genetic technique (Fig 1). The correct construct and the absence of mutations in the inserted gene between the positions of RV-N and RV-M were confirmed using sequencing of the produced viral genome. In the immunofluorescence assay, expression of MERS-CoV S1 protein was confirmed in BHK-P cells infected with RVΔP-MERS/S1 but not in BHK-P cells infected with RVΔP, whereas RV-N-antigen-positive cells were observed in both RVΔP-MERS/S1- and RVΔP-infected BHK-P cells (Fig 2). Moreover, western blot analysis confirmed the existence of the MERS-CoV S1 protein in RVΔP-MERS/S1-infected BHK-P cells but not in RVΔP-infected BHK-P cells, whereas the RV-G protein was detected in both RVΔP-MERS/S1- and RVΔP-infected BHK-P cells (Fig 3a). Furthermore, the MERS-CoV S1 protein was detected in both PEG-precipitated and purified RVΔP-MERS/S1 but not in PEG-precipitated and purified RVΔP, whereas RV-G protein was observed in PEG-precipitated RVΔP-MERS/S1, purified RVΔP-MERS/S1, PEG-precipitated RVΔP, and purified RVΔP (Fig 3b).

### Growth kinetics of RVΔP-MERS/S1 and RVΔP in BHK-P cells

**Fig 4:**
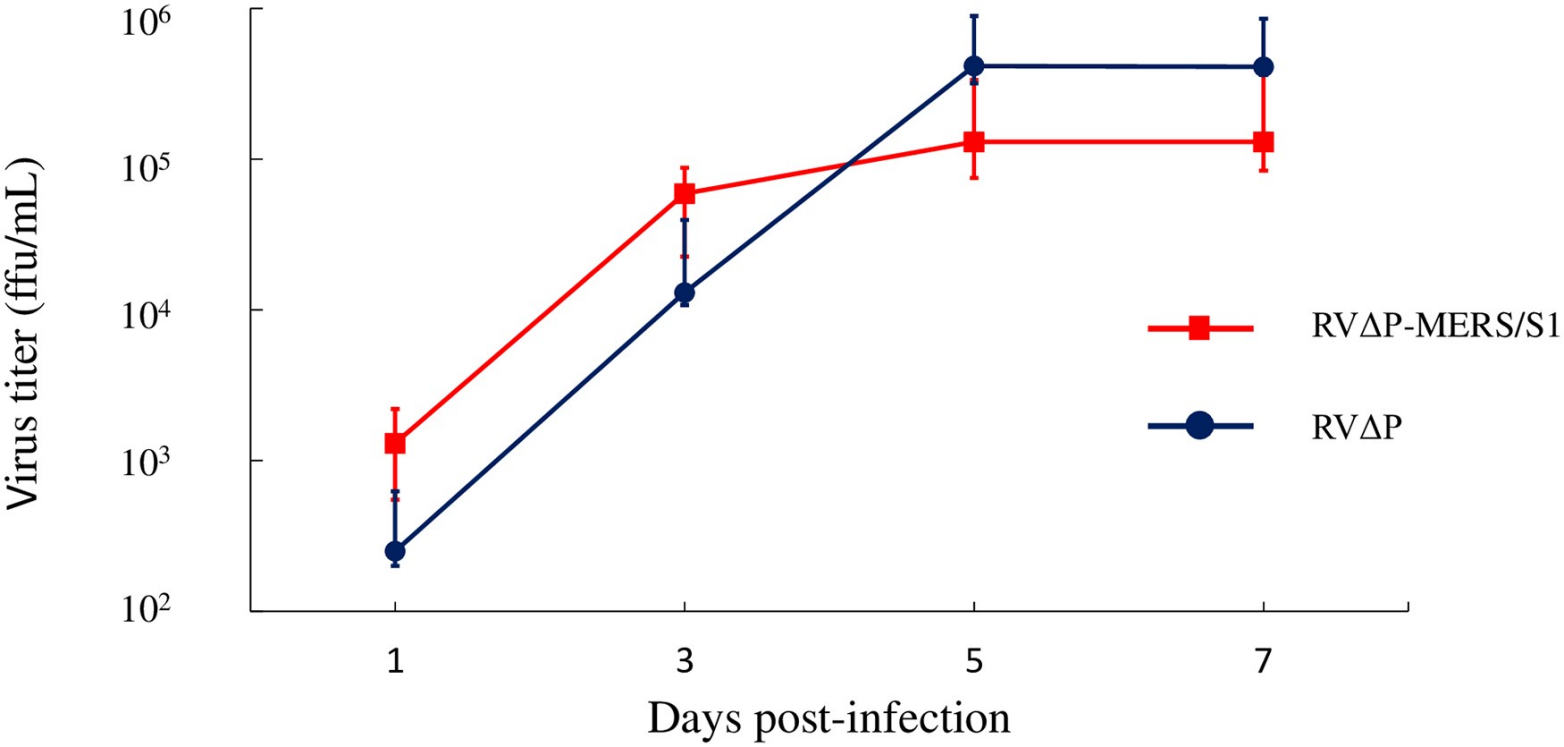
Growth curves of RVΔP-MERS/S1 and RVΔP in BHK-P cells. BHK-P cells were infected with each of the recombinant viruses, RVΔP-MERS/S1 or RVΔP, at a MOI of 0.01/cell. Culture supernatants were harvested on days 1, 3, 5, and 7, and virus titers were determined using BHK-P cells. Titers of viruses were obtained from 3 independent experiments. The Mann-Whitney U test was used, and p<0.05 was considered significant.

BHK-P cells were respectively infected with each of the recombinant viruses (RVΔP-MERS/S1 and RVΔP) at an MOI of 0.01/cell and incubated at 33 °C for 7 days. Titers were determined on days 1, 3, 5, and 7, with the day of inoculation representing day 0 (Fig 4). The growth properties of the recombinant viruses did not differ significantly between RVΔP-MERS/S1 and RVΔP, with the titers of both reaching a peak on day 5 and maintaining that level through day 7 (maximum titer: 10^5^ FFU/mL).

### RVΔP-MERS/S1 and RVΔP pathogenicity in suckling mice

**Fig 5:**
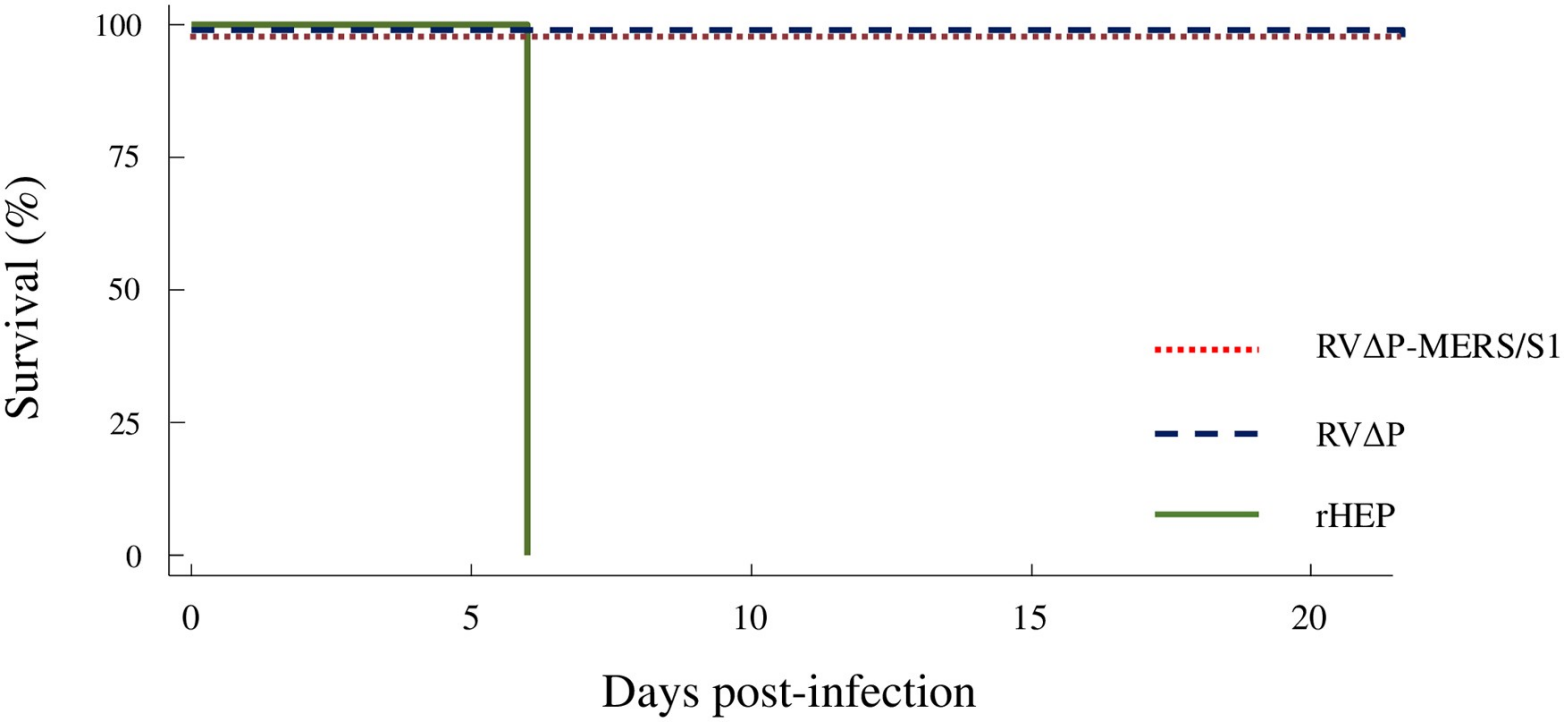
Safety profile of RVΔP-MERS/S1 in suckling mice. Four-day-old suckling mice (n = 16 in each group) were inoculated with RVΔP-MERS/S1 (red), RVΔP (blue), or rHEP (green) through intracerebral inoculation and observed for 3 weeks. All mice were inoculated intracerebrally with 20 μL of virus solutions containing 10^7^ FFU/mL of each virus.

To evaluate the pathogenicity of the recombinant viruses (RVΔP-MERS/S1 and RVΔP), suckling mice were intracerebrally inoculated with RVΔP-MERS/S1, RVΔP, or rHEP, and clinical signs were monitored. No mice inoculated with RVΔP-MERS/S1 or RVΔP showed any rabies-associated signs, and all survived the observation period (Fig 5). On the other hand, seven of the 16 suckling mice inoculated with 2.0 ×10^3^ FFU of rHEP showed sickness after 4-days post-inoculation, and two mice died at 6-days post-inoculation before meeting criteria for euthanasia because of rabies-associated neurological diseases; the other mice were sacrificed immediately after they reached the moribund stage on the same day. Significant differences in survival rates were observed between mice inoculated with rHEP and RVΔP-MERS/S1 or RVΔP, suggesting that not only RVΔP but also RVΔP-MERS/S1 remained non-pathogenic in mice.

### Induction of VNAs against MERS-CoV and RV

**Fig 6:**
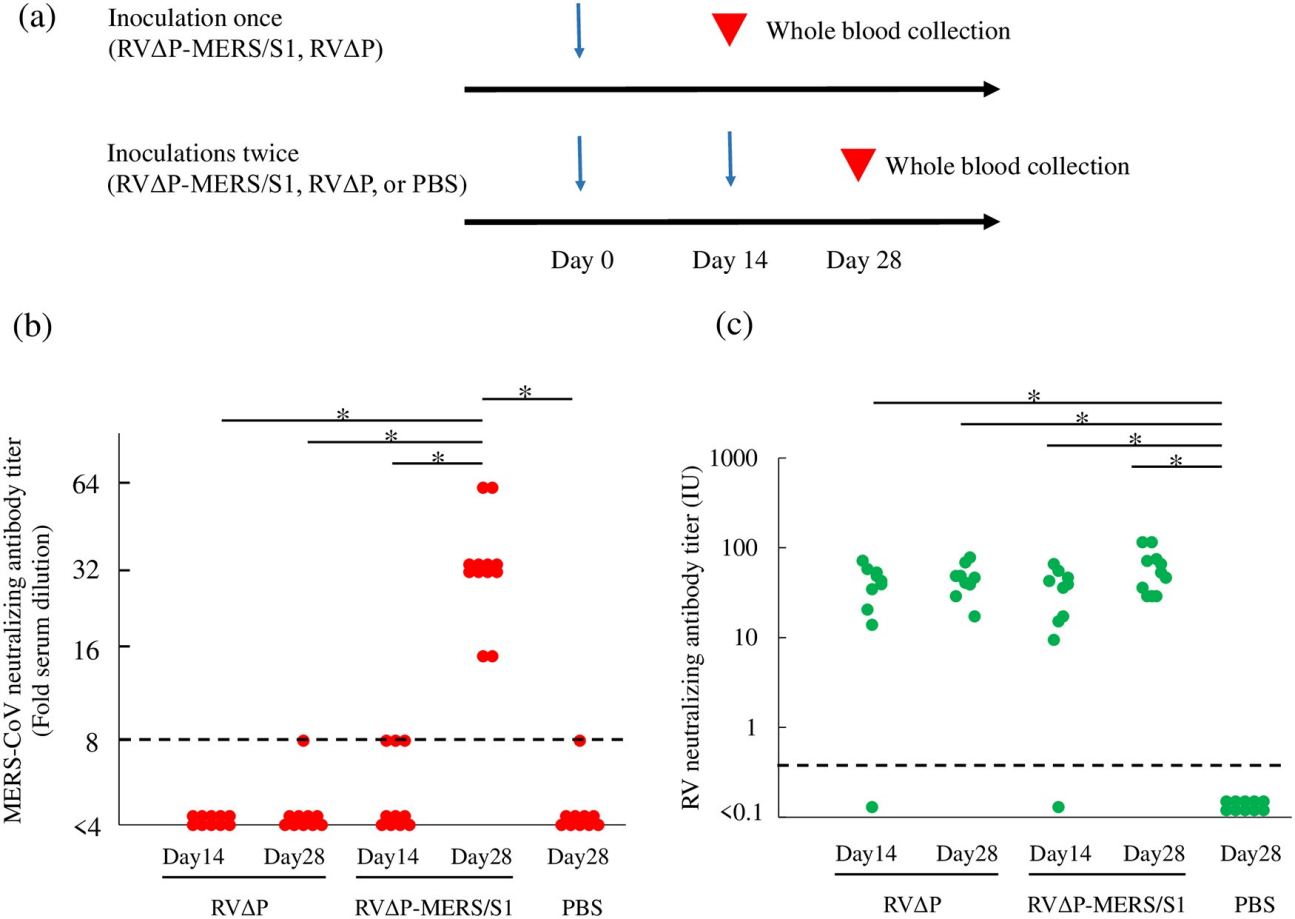
Analysis of VNAs against MERS-CoV and RV in mice inoculated with RVΔP-MERS/S1 or RVΔP. a) Immunization and whole-blood collection schedules of 4-week-old female BALB/c mice. Mice were inoculated with RVΔP-MERS/S1, RVΔP, or PBS as control. All mice were inoculated intraperitoneally with 100 μL of virus solution containing 10^7^ FFU/mL of each virus. Titers of VNAs against MERS-CoV and RV were determined using the serum of mice 14 days after last inoculation. b) Titers of VNAs against MERS-CoV detected in the serum of mice inoculated with RVΔP-MERS/S1 (n = 12 in mice inoculated once or twice, respectively), RVΔP (n = 10 in mice inoculated once or twice, respectively), or PBS (n = 10 in mice inoculated twice). c) Titers of VNAs against RV were also detected in the same samples. The Steel-Dwass nonparametric test was used, and asterisks indicate a significant difference (p < 0.05).

To evaluate the immunogenicity of the recombinant viruses (RVΔP-MERS/S1 and RVΔP), mice were inoculated once or twice with RVΔP-MERS/S1 or RVΔP or twice with PBS (Fig 6a), and titers of VNAs against MERS-CoV and RV were determined using sera from these mice (Fig 6b and 6c). Although VNA titers against MERS-CoV in sera from mice inoculated twice with RVΔP-MERS/S1 were ≥16, those in sera from mice inoculated once with RVΔP were not detected. Additionally, VNA titers in three mice inoculated once with RVΔP-MERS/S1 and one mouse inoculated twice with RVΔP and PBS reached 8-fold dilution, suggesting this titer as background. Significant differences in titers were found between mice inoculated twice with RVΔP-MERS/S1 and the other four groups (Fig 6b). On the other hand, VNA titers against RV were >0.5 IU in sera from almost all mice inoculated with RVΔP and RVΔP-MERS/S1. Moreover, the median VNA titers reached 41.0 IU (range: 0.10–71.9 IU) and 47.7 IU (range: 17.3–78.4 IU) in sera from mice inoculated once or twice with RVΔP, respectively, and 37.6 IU (range: 0.10–65.9 IU) and 49.9 IU (range: 29.0–115.4 IU) in those inoculated once or twice with RVΔP-MERS/S1, respectively, whereas VNA titers in sera from mice inoculated with PBS remained at <0.5 IU. Significant differences were found between mice inoculated with PBS and those with RVΔP and RVΔP-MERS/S1, whereas no significant differences were observed in VNA titers against RV among mouse groups inoculated with RVΔP and RVΔP-MERS/S1 (Fig 6c).

## Discussion

In this study, a recombinant P-gene-deficient RV expressing the MERS-CoV S1 protein, was generated as a bivalent-vaccine candidate to MERS-CoV and RV. Intraperitoneal inoculation of mice twice with RVΔP-MERS/S1 elicited VNAs against both MERS-CoV and RV. These results suggest that a MERS-CoV S1-protein-expressing RVΔP-based vector system is a potential immunogen for MERS-CoV, as well as RV. Additionally, this RVΔP-MERS/S1 vaccine candidate did not kill suckling mice, even when inoculated intracerebrally, thereby exhibiting a complete safety profile according to *in vivo* assays.

Several vaccine platforms against MERS-CoV display immunogenicity, efficacy, and safety in animal experiments [6,47,48]. Of these vaccine platforms, the recombinant viral vector is considered among the most promising. To date, viral vectors utilizing modified vaccinia virus Ankara, measles virus, or adenovirus have been used to express MERS-CoV glycoprotein and shown to be immunogenic according to *in vivo* assays [49–52]. In addition to these recombinant viral vectors, RV vectors are also promising. Recombinant RVs expressing various foreign antigens elicit protective immunity against corresponding pathogens, such as MERS-CoV [15] human immunodeficiency virus type 1 [30], Ebola virus [53], severe acute respiratory syndrome (SARS)-CoV [31], and hepatitis C virus [54].

A previous study demonstrated that neutralizing mAbs significantly reduced virus titers in the lungs of mice infected with MERS-CoV by targeting the RBD of the spike protein [55], indicating that VNAs could play an important role in protecting mice from MERS-CoV infection. In another report, inactivated replication-competent recombinant RV expressing the MERS-CoV S1 protein elicited VNAs and protected mice from MERS-CoV challenge [15]. In the present study, RVΔP-MERS/S1 induced neutralizing activity against MERS-CoV in mice inoculated with RVΔP-MERS/S1. The induction of protective immunity in mice against MERS-CoV infection following immunization with RVΔP-MERS/S1 suggests its potential efficacy in other mammals, including humans. To evaluate the efficacy of vaccines developed against MERS-CoV, various experimental animals ranging from small animals to nonhuman primates have been used for challenge tests [56]. The present study investigated the antibody-inducing capacity of RVΔP-MERS/S1 in mice; however, further animal-challenge tests are required to evaluate whether RVΔP-MERS/S1 can induce protective immunity against MERS-CoV.

The WHO-mandated VNA level against RV of 0.5 IU/mL is widely used as an indication of adequate vaccination [57]. In the present study, RVΔP-MERS/S1 induced significantly high-titer VNAs against RV. Given the correlation between VNAs and protection, it is expected that this RVΔP-MERS/S1 vaccine candidate will confer protection against RV.

Generally, viral vectors are highly effective; however, there exists the possibility of safety concerns. In this respect, our replication-deficient recombinant RV-based vector (RVΔP) is highly advantageous, given that RVΔP-vector vaccines are completely non-pathogenic in animals due to the inability of RVΔP to replicate in infected hosts [26,29]. Moreover, RVΔP-MERS/S1 vaccination did not cause disease in suckling mice, suggesting the safety of this vaccine candidate. Deletion of the gene encoding the P protein from the genome of RVΔP-MERS/S1 precluded the possibility of reversion to a virulent virus [22], with this characteristic of the replication-deficient vaccine candidate promoting its efficacy for use as a live vaccine. A previous study reported the avirulence of a replication-deficient RV virus expressing Ebola virus glycoprotein according to the survival of all adult and suckling mice intracerebrally inoculated with the virus along with maintenance of intact brain architecture [58]. By contrast, a replication-competent Ebola virus glycoprotein-expressing RV vaccine recovered from the SAD B19 RV wildlife vaccine strain and harboring an attenuating point mutation retained its neurovirulence in suckling mice when administered intracerebrally [58,59].

In this study, MERS-CoV S1 subunit expressed by RVΔP-MERS/S1 was constructed to be translated together with the RV-G transmembrane and cytoplasmic domains and 14 amino acids of the ectodomain of RV-G. There were several reasons to use this construct. First, because of the weak capacity for transcription and replication of the P-deficient RV vector in cells, it was expected that the full-length S gene (~4 kb) was too long to express using the RVΔP vector. In fact, we found that a recombinant virus containing the S gene of MERS-CoV with the deletion of C-terminal 16 amino acids was rescued but did not express the S1 protein with the variable deletion of S1 gene in the genome (S1 Fig), presumably suggesting that this fragment was too large to be inserted into the RVΔP vector. Second, the transmembrane and cytoplasmic domains of RV-G were fused with the S1 protein, because these regions play an important role in the expression of foreign proteins on the surface of virions [60]. Interestingly, there appears to be a loss of S1 protein after purification with sucrose gradient compared with that of PEG-precipitated viruses (Fig 3b); nevertheless, S1 was fused with transmembrane and cytoplasmic domains of RV for surface expression. Presumably, the specific S1 protein might be produced in the cell line, but it might be incorporated onto the surface partially because the original RV-G protein would be easier to incorporate into the virions and be more dominant than the foreign proteins. Finally, insertion of the S1 subunit is reportedly more effective than insertion of the full-length S gene for the development of DNA vaccines for MERS-CoV [61]. In the case of the adenovirus 5 (Ad5) vector-based vaccine, MERS-CoV S1-subunit-expressing Ad5 induced stronger VNA responses than Ad5 expressing the full-length S protein [62,63]. This might be because immunization with the S1 subunit can induce humoral immune responses more efficiently than that with the full-length S protein [62]. Additionally, there might remain concerns regarding the safety of using vaccines that express the full-length S protein, as such vaccines can potentially induce harmful side effects caused by non-neutralizing epitopes [62]. A previous SARS-CoV vaccine study reported inflammatory and immunopathological effects, such as eosinophilic infiltration, in immunized animals [64]. Therefore, insertion of the S1 subunit might be more favorable as an antigen relative to using the full-length S protein for vaccine development.

As described in the introduction, MERS and rabies have resulted in many fatal cases, suggesting that these diseases remain a serious threat to global public health. Sero-epidemiological studies demonstrated the presence of MERS-CoV in dromedaries in the Middle East and Africa [65,66], where rabies epidemics have occurred. Considering this epidemiological finding, a bivalent vaccine, such as RVΔP-MERS/S1, against MERS and rabies would be useful for its practical application to control such situations.

In conclusion, we found that intraperitoneal vaccination with RVΔP expressing the MERS-CoV S1 protein induced VNAs against MERS-CoV and RV in mice, suggesting RVΔP-MERS/S1 as a promising bivalent-vaccine candidate against MERS and RV. This study builds upon our previous work describing the usefulness of an RVΔP-based vaccine against lymphocytic choriomeningitis virus and supports further application of RVΔP-based bivalent-vaccine development against rabies and other infectious diseases.

## Supporting information

S1 Fig **(a) Schematic illustration of RVΔP-MERS/St16**. RVΔP-MERS/St16 harbors the MERS-CoV S1+S2 gene with the C-terminal 16 amino acids deleted (amino acids 1 to 1337) between RV-N and RV-M genes of the RV genome. **(b) Immunofluorescence staining of RV-N and MERS-CoV S1 protein expression of RVΔP-MERS/St16**. BHK-P cells were inoculated with either RVΔP-MERS/St16 or RVΔP at an MOI of 0.1/cell and incubated at 33°C for 48 h. Cells were stained with the monoclonal antibody against RV-N (green) or the monoclonal antibody MERS-CoV S1 protein (red), respectively. Cells were observed with a fluorescence microscope OLYMPUS X-81 (Olympus, Tokyo, Japan). Images were acquired with an ORCA-R2 (Hamamatsu Photonics K.K., Shizuoka, Japan) and colored with LuminaVision (MITANI Corporation, Tokyo, Japan).
(TIF)
