# Supplementary figures and images for: Development of a recombinant replication-deficient rabies virus-based bivalent-vaccine against MERS-CoV and rabies virus and its humoral immunogenicity in mice
Source: PLoS One. 2019 Oct 7;14(10):e0223684. doi: 10.1371/journal.pone.0223684 (PMC6779238; doi:10.1371/journal.pone.0223684)

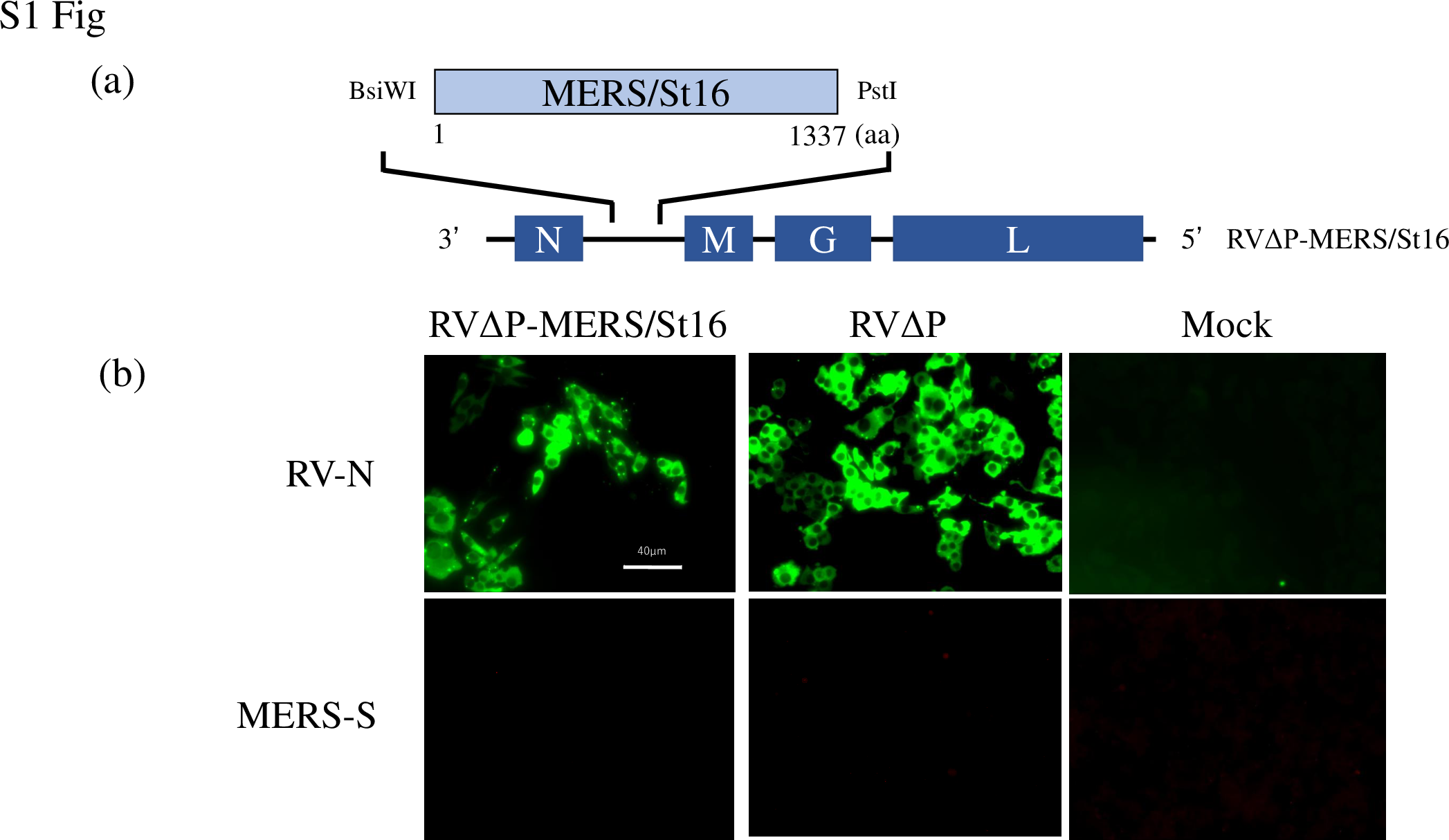

Supplement: S1 Fig — (a) Schematic illustration of RVΔP-MERS/St16. RVΔP-MERS/St16 harbors the MERS-CoV S1+S2 gene with the C-terminal 16 amino acids deleted (amino acids 1 to 1337) between RV-N and RV-M genes of the RV genome. (b) Immunofluorescence staining of RV-N and MERS-CoV S1 protein expression of RVΔP-MERS/St16. BHK-P cells were inoculated with either RVΔP-MERS/St16 or RVΔP at an MOI of 0.1/cell and incubated at 33°C for 48 h. Cells were stained with the monoclonal antibody against RV-N (green) or the monoclonal antibody MERS-CoV S1 protein (red), respectively. Cells were observed with a fluorescence microscope OLYMPUS X-81 (Olympus, Tokyo, Japan). Images were acquired with an ORCA-R2 (Hamamatsu Photonics K.K., Shizuoka, Japan) and colored with LuminaVision (MITANI Corporation, Tokyo, Japan). (TIF) [file pone.0223684.s001.tif]
